# Paleobiology of the Latest Tithonian (Late Jurassic) Ammonite *Salinites grossicostatum* Inferred from Internal and External Shell Parameters

**DOI:** 10.1371/journal.pone.0145865

**Published:** 2016-01-13

**Authors:** Patrick Zell, Wolfgang Stinnesbeck

**Affiliations:** Institut für Geowissenschaften, Universität Heidelberg, Heidelberg, Germany; Naturhistoriska riksmuseet, SWEDEN

## Abstract

Based on material from the uppermost Tithonian La Caja Formation at Puerto Piñones, northeastern Mexico, the complete ontogenetic development (protoconch to adult) of the ammonite *Salinites grossicostatum* is outlined by a detailed morphometrical shell analysis. The embryonic stage, consisting of a small ellipsoid protoconch and ammonitella, ends at about 0.6 mm. Four major morphological changes are differentiated throughout ontogeny based on internal features such as reduced septal spacing and siphuncle position. Sexual dimorphism is reflected by shell size, siphuncular diameter, differences in the morphology of the apophysis, and by two distinct general trends in septal spacing. In addition, macroconchs are characterized by septal crowding at different stages, followed by the return to normal septum distances. Our analysis indicates a change in the mode of life after the neanic stage. A change in habitat preference is inferred for adult individuals. While microconchs persisted at Puerto Piñones, large mature macroconchs temporarily migrated to other areas, possibly for egg deposition. *Salinites grossicostatum* is endemic to the ancient Gulf of Mexico and is there restricted to outer continental shelf environments.

## Introduction

Ammonite morphology and internal structures yield important information on both the systematic position of species as well as their paleobiology (e.g. dimorphism, habitat preferences, migration during sexual maturity, responses to seasonality), but only a small number of taxa has been analyzed in detail by using a combination of external and internal sets of data (e.g. [1]). In consequence, the complete shell ontogeny of ammonites from embryonic to late post-embryonic stages is little known to date. For instance, an analysis of septal spacing in combination with shell diameter and ornamentation data revealed a gradual decrease in septum distances in adults reaching maturity (e.g. [2]), while septal crowding was regarded as rare in premature individuals [2]. In other taxa septal crowding was attributed to adverse ecological circumstances, or injuries [2–6].

Here we provide a detailed analysis of the ontogenetic development of the Late Jurassic ammonite *Salinites grossicostatum* (Imlay 1939) [7], combining data on external morphology with internal characters such as septal spacing and angles, suture and siphuncular position and size. Our data allow us to differentiate sexual dimorphism and four stages of growth. We discuss the implications of these data for the interpretation of life span, habitat preferences, seasonal environmental changes and migration of mature macroconchs.

## Materials and Methods

**Fig 1:**
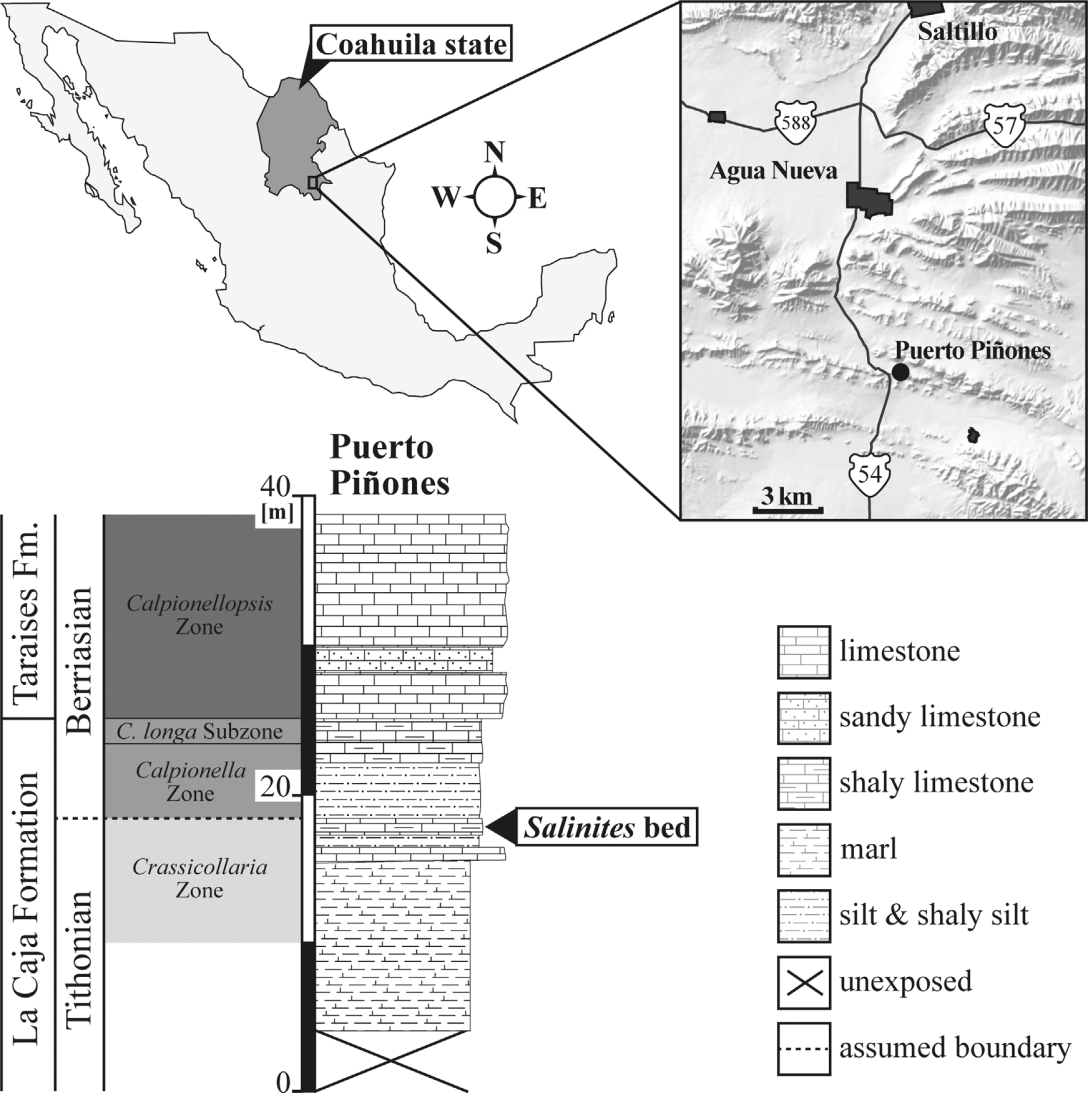
Map of Mexico with inset of northeastern Mexico and the Jurassic-Cretaceous transition at Puerto Piñones. *Salinites grossicostatum* specimens described here originate from a shaly limestone of the latest Tithonian “*Kossmatia-Durangites-Salinites* Beds” (cf. [12]), respectively the uppermost *Crassicollaria* Zone [8]. Locality map after [13]. Puerto Piñones: N25°02.719’/W101°03.396’.

The specimens described here are from Coahuila, northeastern Mexico, and were collected at Puerto Piñones from a 0.3 m-thick layer of shaly limestone of the topmost La Caja Formation (Fig 1) [8–11]. This limestone was assigned by Adatte et al. [8] to the uppermost Tithonian “*Durangites* beds”, respectively the upper *Crassicollaria* Zone, and is stratigraphically located only a few mm below the Jurassic–Cretaceous boundary based on calpionellid occurrences [8]. The ammonite fauna is relatively diverse and is numerically dominated by *Salinites grossicostatum*, while *Pronites*, *Himalayites*, *Kossmatia* and *Durangites* are also present, but only sum up to 5–10% of total individuals. For the analysis of internal morphology and structures, six specimens of *S*. *grossicostatum* were polished with silicon carbide powders.

**Fig 2:**
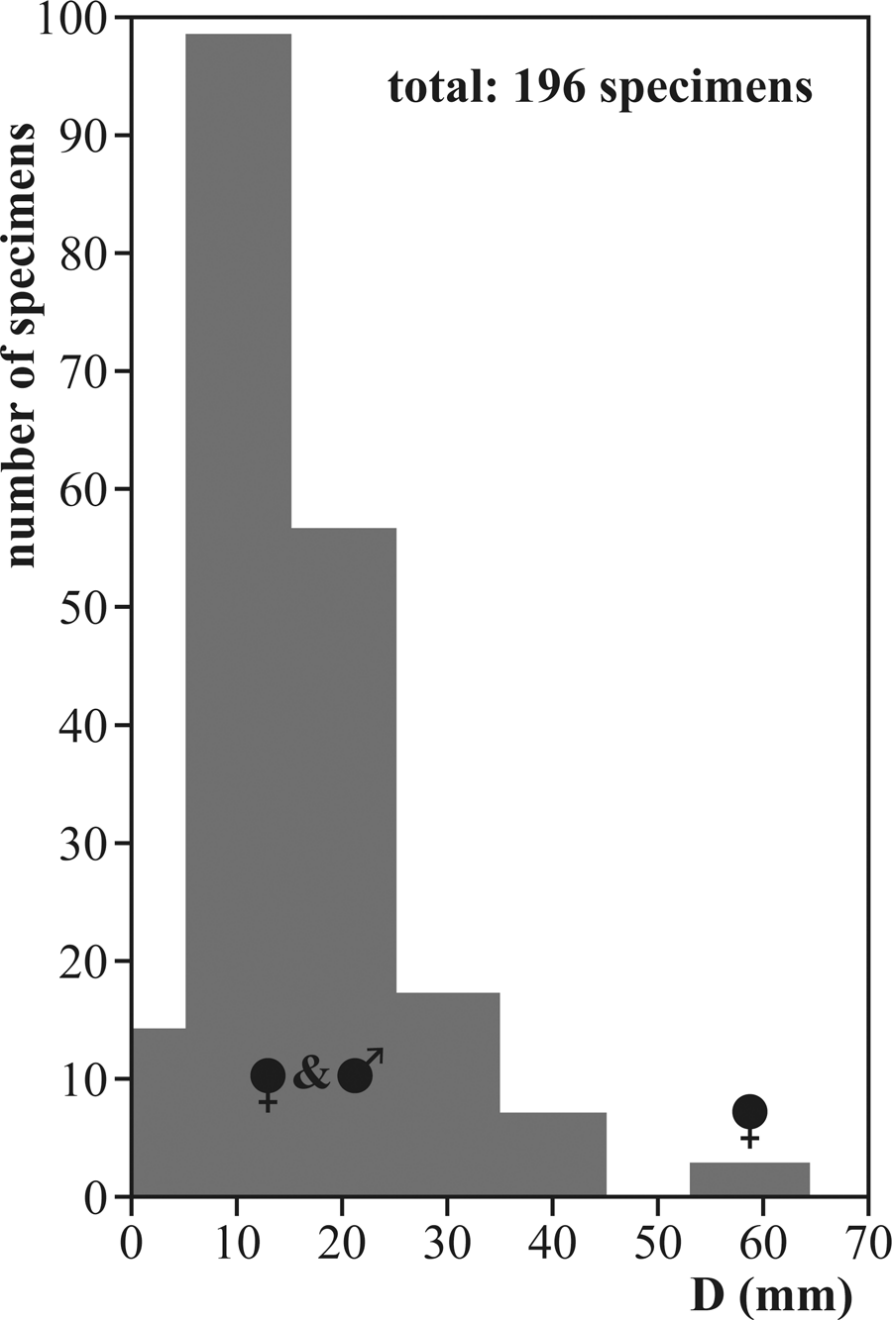
Size-frequency histogram for *Salinites grossicostatum*. Specimens were collected from a single layer of the uppermost Tithonian La Caja Formation at Puerto Piñones. D = diameter.

Our collection of *Salinites grossicostatum* consists of 169 specimens (Fig 2) preserved three-dimensionally (CPC-1214–1242; sample box CPC-1243, CPC-1405–1413). They were collected by Wolfgang Stinnesbeck between 1986 and 1994. No permits were required to access the sampling site at Puerto Piñones. Logistic support and export licenses were provided by the Facultad de Ciencias de la Tierra of the Universidad Autónoma de Nuevo León.

*Salinites grossicostatum* is well preserved and suturelines are locally visible. The body chamber is completely preserved in most specimens and a long apophysis is present in eight individuals. Descriptive terms are those of Arkell et al. [14], Verma & Westermann [15] and Wright et al. [16], and the ontogenetic description follows Neige ([17], and references therein). Abbreviations for ammonites: D, diameter; Wh, whorl height; Ww, whorl width; U, umbilical diameter; U/D, umbilical ratio; Ww/Wh, whorl height to width ratio. Specimens analyzed here are deposited in the Colección de Paleontología de Coahuila (CPC-1214–1242; sample box CPC-1243, CPC-1405–1413) of the Museo del Desierto, Carlos Abendrop Dávila No. 3745, Parque Las Maravillas, Saltillo, Coahuila C.P. 25015, Mexico (http://www.museodeldesierto.org).

### The latest Tithonian *Salinites grossicostatum*

The latest Tithonian [8] *Hildoglochiceras grossicostatum* was first described by Imlay [7] and was assigned to the genus *Salinites* by Cantú-Chapa ([18], p. 19).

**Fig 3:**
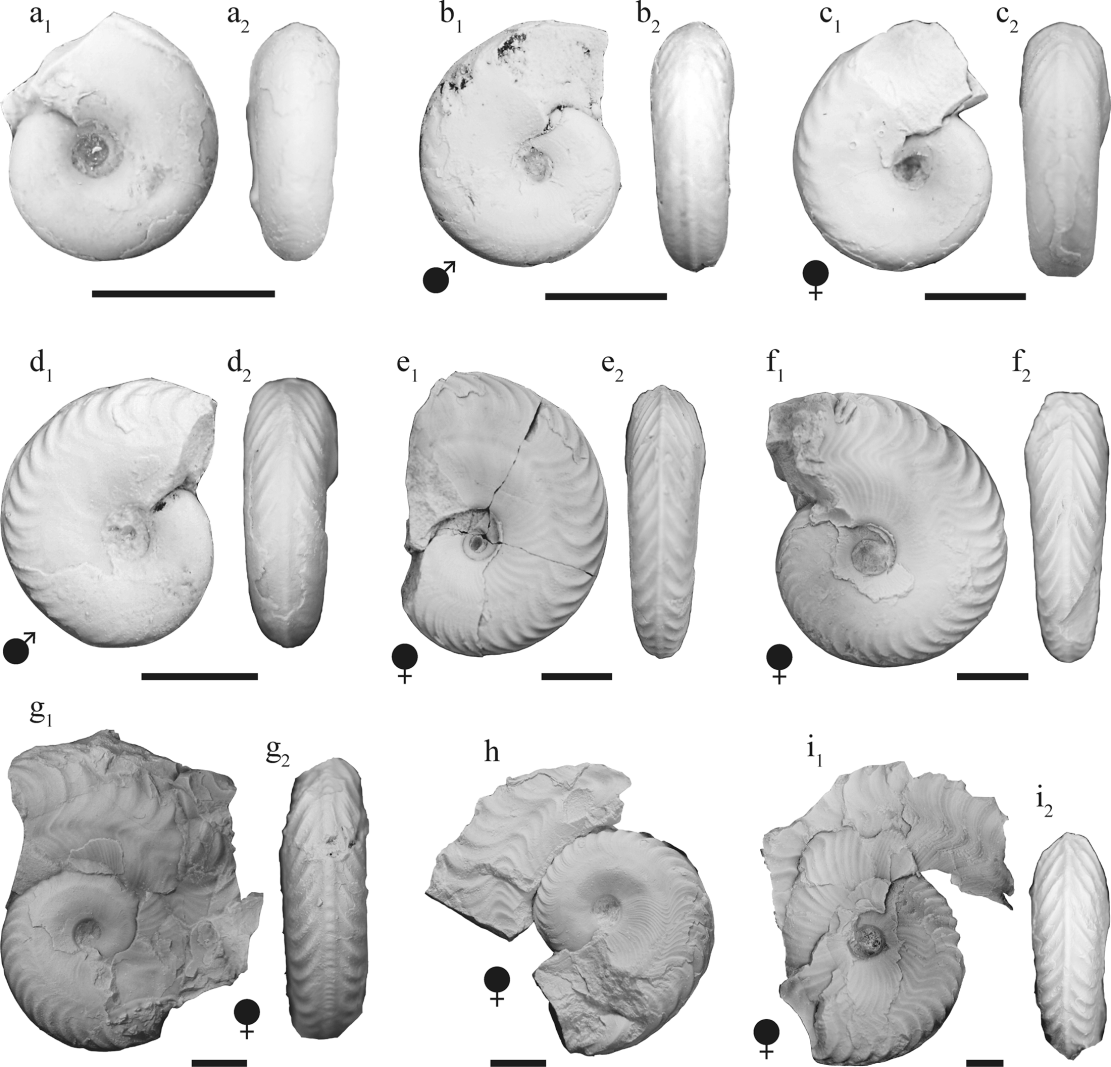
Representative specimens of *Salinites grossicostatum* representing an ontogenetic series from juvenile (a) to mature (i). (a) CPC-1405; (b) CPC-1406; (c) CPC-1407; (d) CPC-1408; (e) CPC-1409; (f) CPC-1410; (g) CPC-1411; (h) CPC-1412; (i) CPC-1413; scale bars = 10 mm.

*Salinites grossicostatum* (Fig 3) is a medium-sized (D_max._ = 63 mm, specimen CPC-1413), involute, discoidal ammonite with largest whorl width dorsally close to the middle of the flank (Ww/Wh = 0.52 to 0.76). A shallow concentric whorl depression is present in the dorsolateral region of the flank. The umbilicus is narrow (U/D = 0.15 to 0.2) and shallow, while the umbilical wall is almost vertical. The flanks are faintly convex in juveniles, and convexity decreases further during later ontogenetic stages. The venter is initially narrowly rounded (Fig 3A) but is successively attenuated and a smooth keel is developed at D≈8.6 mm. The keel becomes serrated at diameters of about 11.2 to 15.5 mm (Fig 3G) and is flanked on both sides by a shallow ventral furrow. Fine ventrolateral ribs first appear at diameters of about 6.4 mm. They are rursiradiate on the middle of the flank and become prorsiradiate towards the venter. From a diameter of about 10 mm onward, fine biconcave growth lines appear between the umbilical ridge and mid-flank; these are first seen on internal moulds but from diameters >15 mm onward are also identified on the shell. These growth lines gradually merge into more prominent ribs during late ontogenetic stages. A shallow spiral groove appears at about mid-flank at diameters of about 9.2 to 13.1 mm; it is first visible on internal moulds but is also seen on the outer shell at diameters >18 mm. These ribs are present to the last preserved whorl of adult specimens. An apophysis develops at about mid-flank at diameters of 19.5 to 25.3 mm. Initially, the apophysis follows the coiling of the shell but it projects ventrolaterally during the final ontogenetic stage of macroconch development. 33 ventrolateral ribs are present on the outermost whorl of microconchs corresponding to 42 mm diameter (CPC-1223), while macroconchs have 37 ventrolateral ribs at 38 mm diameter (CPC-1410). The body chamber of adult specimens in both micro- and macroconchs exhibits all sculptural elements described above, although ribbing on the body chamber is more wavy and irregular. At the apophysis, the ornamentation of macroconchs initially increases in strength but smoothens out again on the last 35° bend behind the terminal aperture (Fig 3H, Fig 3I). The largest specimen reaches a diameter of 64 mm (CPC-1413); in this specimen, the body chamber occupies 2/3 of the last whorl.

An explicit sexual dimorphism is indicated by differential growth rates and internal growth features as detailed below, indicating that the assemblage consists of roughly equal numbers of micro- and macroconchs. The largest specimens (D = 53–63 mm) are here interpreted to represent macroconchs, while no individuals with shell diameters between 45 and 53 mm were found. This bimodal distribution pattern (Fig 2) may indicate changing habitat preferences of female adults during sexual maturity.

*Salinites grossicostatum* is endemic to the ancient Gulf of Mexico, where it is restricted to shallow marine, outer shelf environments at the transition between the continental platform and slope [7]–[8], [18–24].

### Ontogeny of *Salinites grossicostatum*

#### Embryonic development

**Fig 4:**
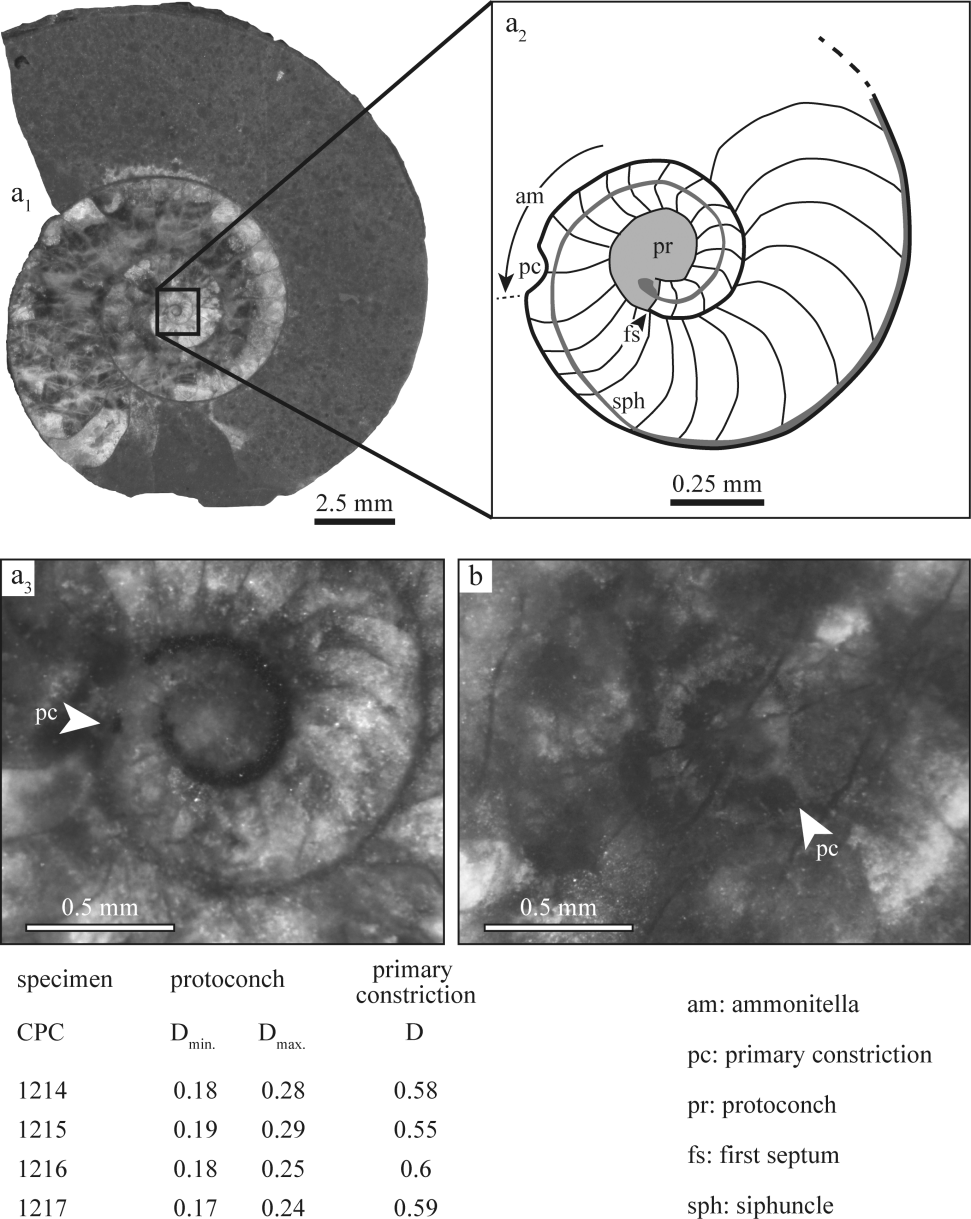
Early ontogenetic internal shell development and measurements of *Salinites grossicostatum* based on four median sections. (a) CPC-1217, with sketch to the right (a_2_) to illustrate internal shell structures; (b) CPC-1214; measurements are provided in mm.

The embryonic ammonoid, termed ammonitella [25], consists of the protoconch (initial chamber) and approximately one spiral whorl initiating at the caecum and terminating at the primary constriction [6], [17], [25]. In *Salinites grossicostatum*, the center of the protoconch (*sensu* [17]) was used to determine the diameter of protoconch and ammonitella (Fig 4A_2_). The mean minimum diameter of the protoconch is 0.18 mm and its mean maximum diameter is 0.265 mm (Fig 4). This considerable variation (68%) is due to the subellipsoidal form of the protoconch. The mean diameter of the ammonitella is 0.58 mm (Fig 4). The ratio between the diameters of ammonitella and maximum protoconch values averages 2.15.

### Apertural height

**Fig 5:**
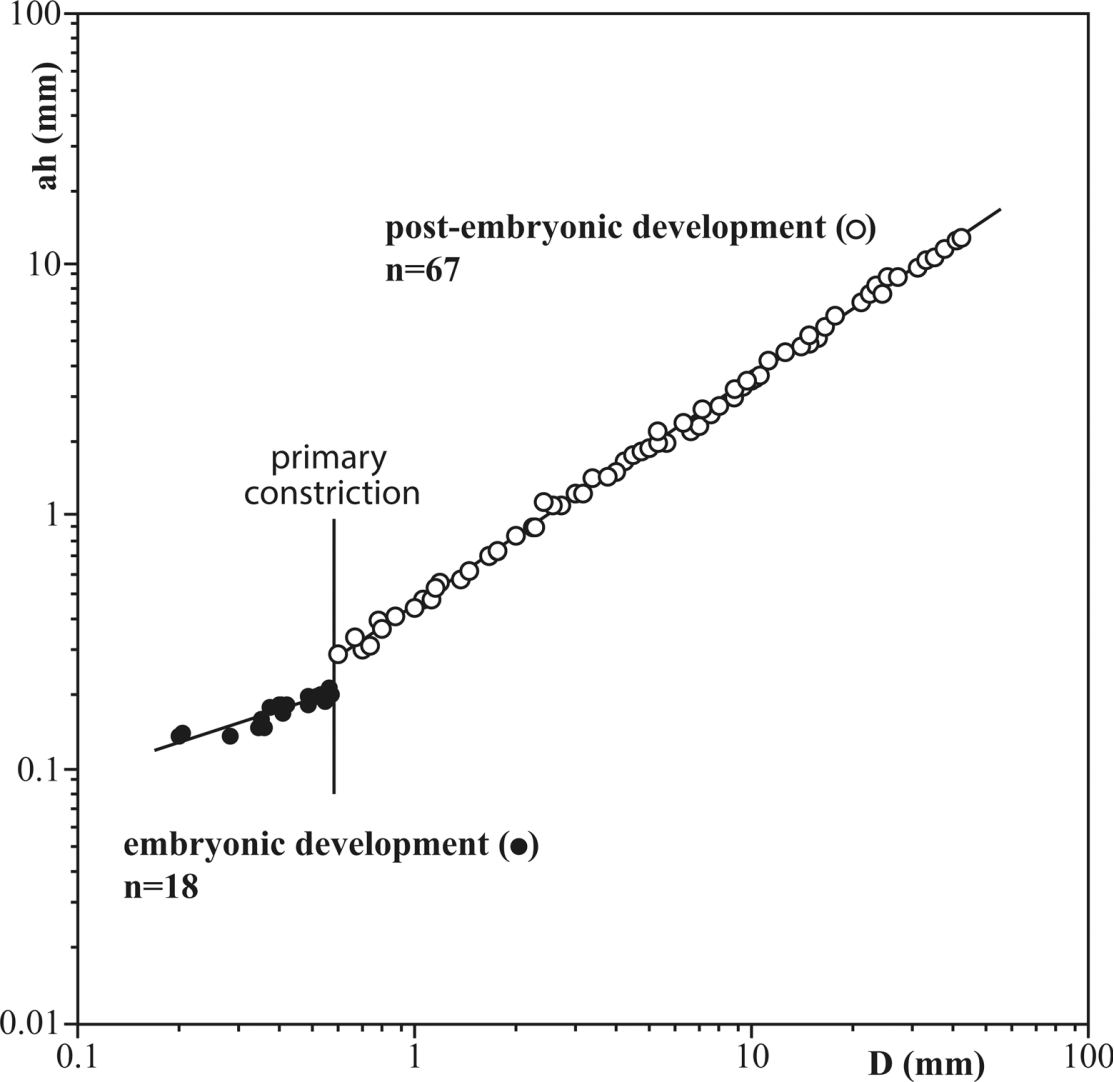
Apertural height (ah) versus shell diameter (D) for two micro- and two macroconchs (CPC-1214–1217). Note that apertural height is illustrated logarithmically. The regression lines indicate a change in growth at the primary constriction and that allometric growth is higher during post-embryonic stages than before.

Apertural heights and their corresponding diameters were measured in four specimens from the protoconch to the shell opening at the end of the last whorl. Results are illustrated in Fig 5 as a logarithmic ratio between the apertural height and shell diameter. Our measurements reveal a small but significant break in growth between the embryonic and post-embryonic stages, coincident with the primary constriction. Our data also indicate that allometric growth was higher in the post-embryonic development than in the initial ontogenetic stage considered as embryonic.

### Siphuncle

**Fig 6:**
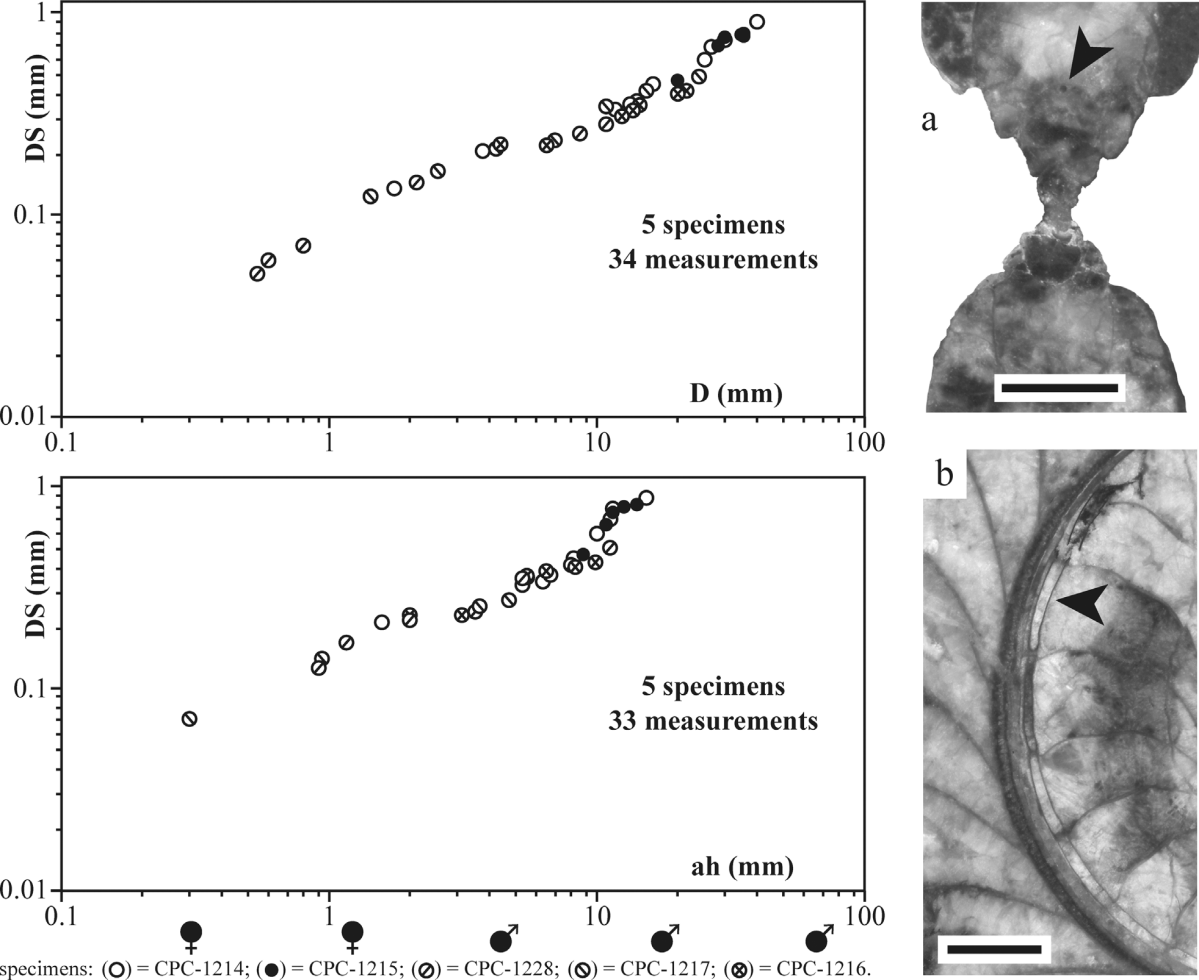
Measurements of the siphuncle in five specimens. The upper plot illustrates the ratio between siphuncular diameter [DS] versus shell diameter [D]; the lower plot figures the siphuncular diameter versus apertural height at the plane of coiling [ah]. To the right, high-resolution scans illustrate the siphuncle; (a) longitudinal median section of CPC-1228; (b) transversal section through the plane of coiling in CPC-1214. Black arrows indicate position of the siphuncle; scale bars = 2.5 mm.

The siphuncular diameter was measured in five specimens (CPC-1214–1217 and 1228; Fig 6). Septa are mostly straight but are slightly incised concavely at the position of the septal neck. The latter is retrochoanitic during the earliest ontogenetic stages (Fig 4A_2_) and becomes prochoanitic (Fig 6B) at D≈1.8 mm, after approximately 18 septa and in the first half of the second whorl (in CPC-1214). The connecting ring is thin and concave. The siphuncle is initially located in a subcentral position but gradually shifts to a ventral position shortly after the primary constriction (Fig 4A_2_). The siphuncle resumes its final ventral position at D≈0.77 mm. Due to recrystallization and partial internal fragmentation, the siphuncle is only partially preserved in CPC-1214–1217 and 1228 (Fig 6B). However, it is well-preserved in the two specimens CPC-1214 and 1228, with a minimum siphuncular diameter of 0.05 mm at D = 0.56 mm (CPC-1228). A maximum diameter of 0.9 mm was reached at the last septum (end of the phragmocone) of macroconch CPC-1214 at D = 40.9 mm (postembryonic stage). The siphuncular diameter gradually increases in size and reveals a uniform first stage up to D≈28 mm (ah≈10 mm). In macroconchs this stage is followed by an abrupt increase in siphuncular diameter. In general, however, the increase of the siphuncular diameter is slower than that of the increase in shell diameter and apertural height, thus indicating a negative allometry.

### Septal analysis

**Fig 7:**
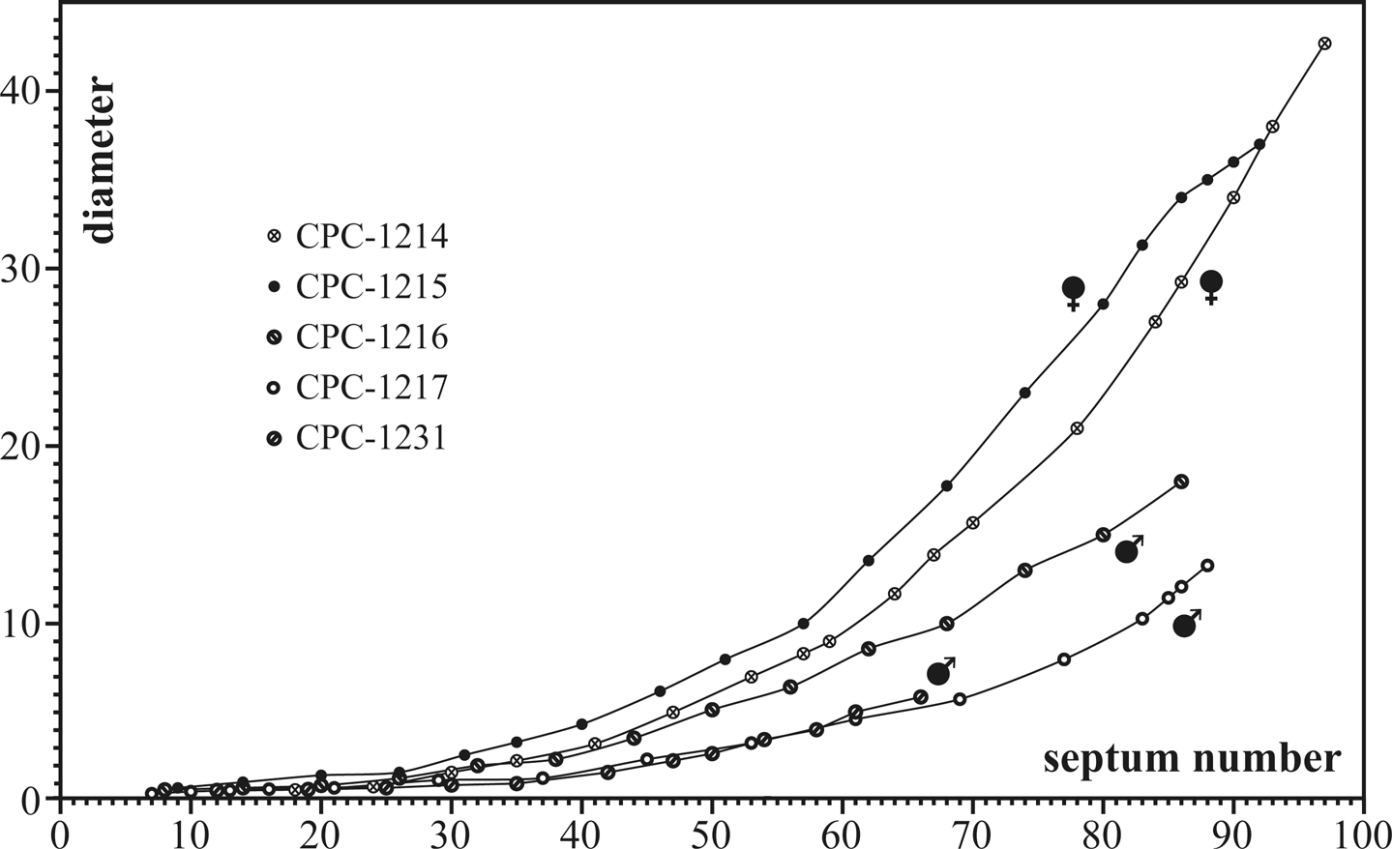
Plot of shell diameter [mm] versus septum number for five specimens. Sexual dimorphism is indicated between septum numbers 25 and 30 by a considerably higher growth increase in macroconchs.

The complete number of septa was analyzed in four adult specimens (CPC-1214–1217) and one juvenile (CPC-1231; Fig 7). The number of septa in the four adult individuals reaches a maximum of 97 and shows no major variation (maximum between 86 and 97), even though the two adult microconchs (CPC-1216 and 1217) are considerably smaller (D = 11 mm, CPC-1217; D = 18.7 mm, CPC-1216) than the macroconchs (D = 42.8 mm, CPC-1214; D = 37.6, CPC-1215). The number of septa thus appears to be genetically confined to a maximum of about 100 in both micro- and macroconchs. Fig 7 illustrates the correlation between the number of septa and the diameter of *S*. *grossicostatum*. The general shape of these graphs is similar in the five individuals analyzed and reveals a uniform first stage of ontogenetic development, up to septum numbers of 25 to 30. Subsequently, however, the growth rates of micro- and macroconchs differ substantially (Fig 7). The end of growth in specimen CPC-1215 is marked by an inflection.

**Fig 8:**
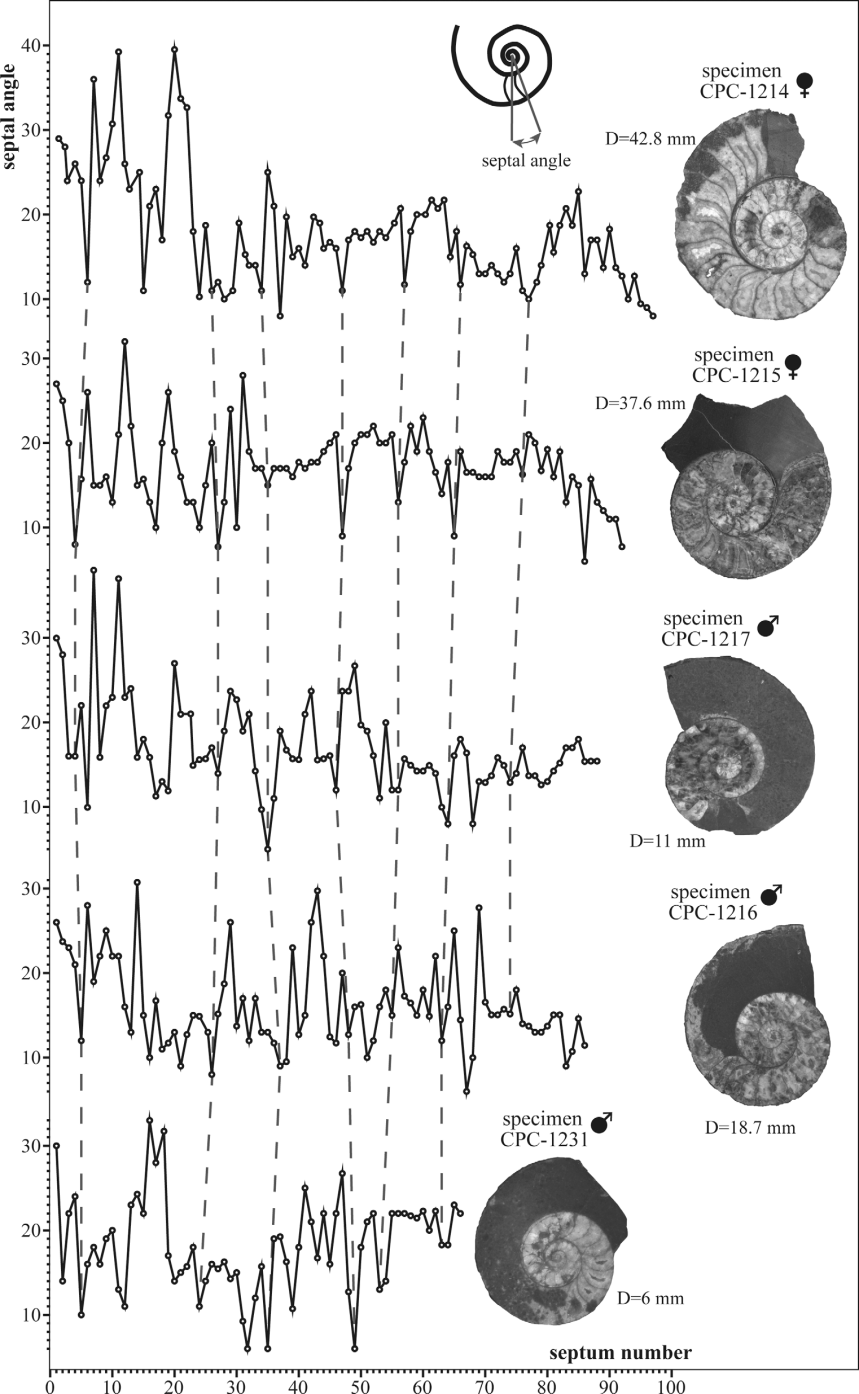
Ratio between septal angle versus septal number for four mature specimens and one juvenile (CPC-1231). Specific correlative septal “events” are marked by dashed lines. D = diameter at the last septum.

The interseptal angle, which corresponds to interseptal spacing, varies during ontogeny (Fig 8). Three general phases are indicated in mature specimens (CPC-1214–1217). Specific septal “events” are detected here and are indicated, in Fig 8, by dashed lines.

Each trajectory initiates with a stable stage of strong septal angle variations, with widest angles reaching 39.4°. This stage ends after 20 to 23 septa and is followed by a stable stage in macroconchs (CPC-1214 and 1215), and a relatively unstable stage in microconchs (CPC-1216, 1217, 1231), with the majority of angles of about 16.6° but also including extremely low angles of 5°. This stage ends after approximately 63 septa. The final growth stage (64 septa and more) is characterized by septal crowding with angles ranging between 6° (CPC-1215) and 18.3° (CPC-1214), with an average of about 12.7°.

### Suture line

**Fig 9:**
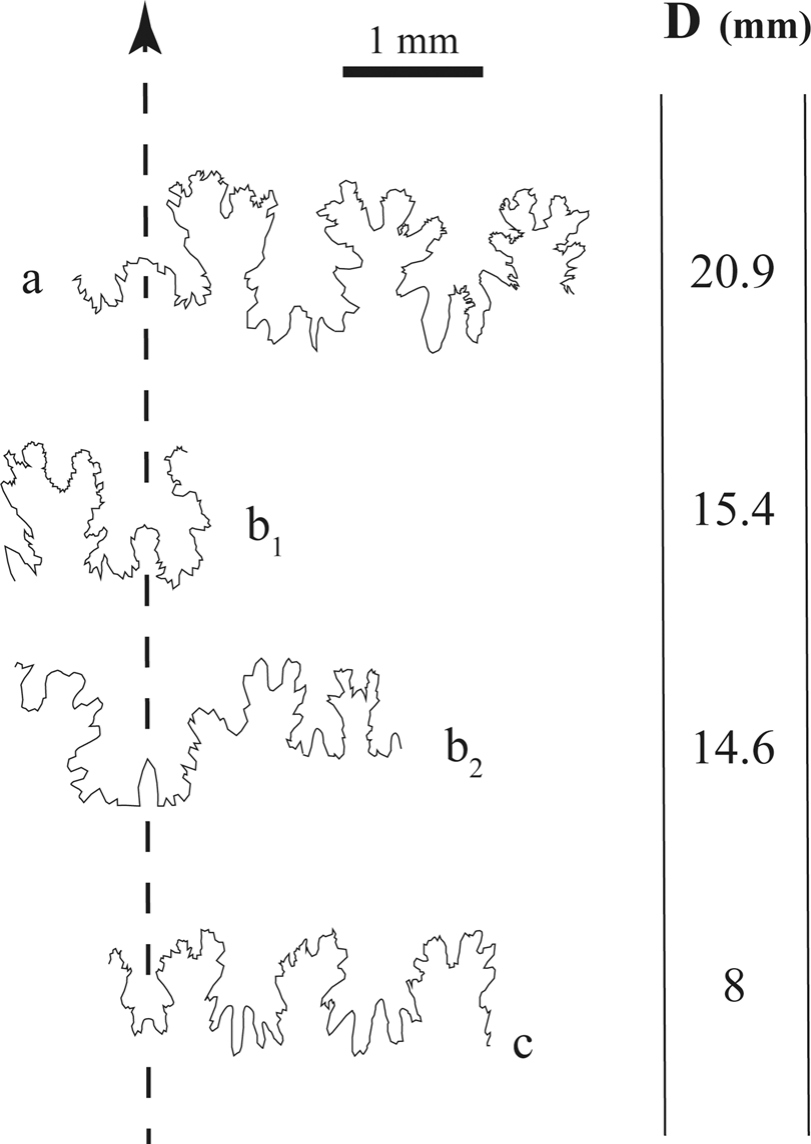
Sutural ontogeny of *Salinites grossicostatum*. (a) CPC-1218; (b) CPC-1219; (c) CPC-1220.

Suture lines are only fragmentarily visible in three specimens (Fig 9). In the early post-embryonic stage (D = 8 mm; Fig 9C), the largest visible saddle is the third lateral, while the first lateral is smaller. The largest lobe is the second lateral. The ventral lobe is simple and small, higher than wide. The ventral saddle becomes larger and wider towards late ontogenetic stages. At D = 20.9 mm, the ventral saddle is subrectangular and furcated, while lateral lobes and saddles are stronger furcated.

### Ornamentation

**Fig 10:**
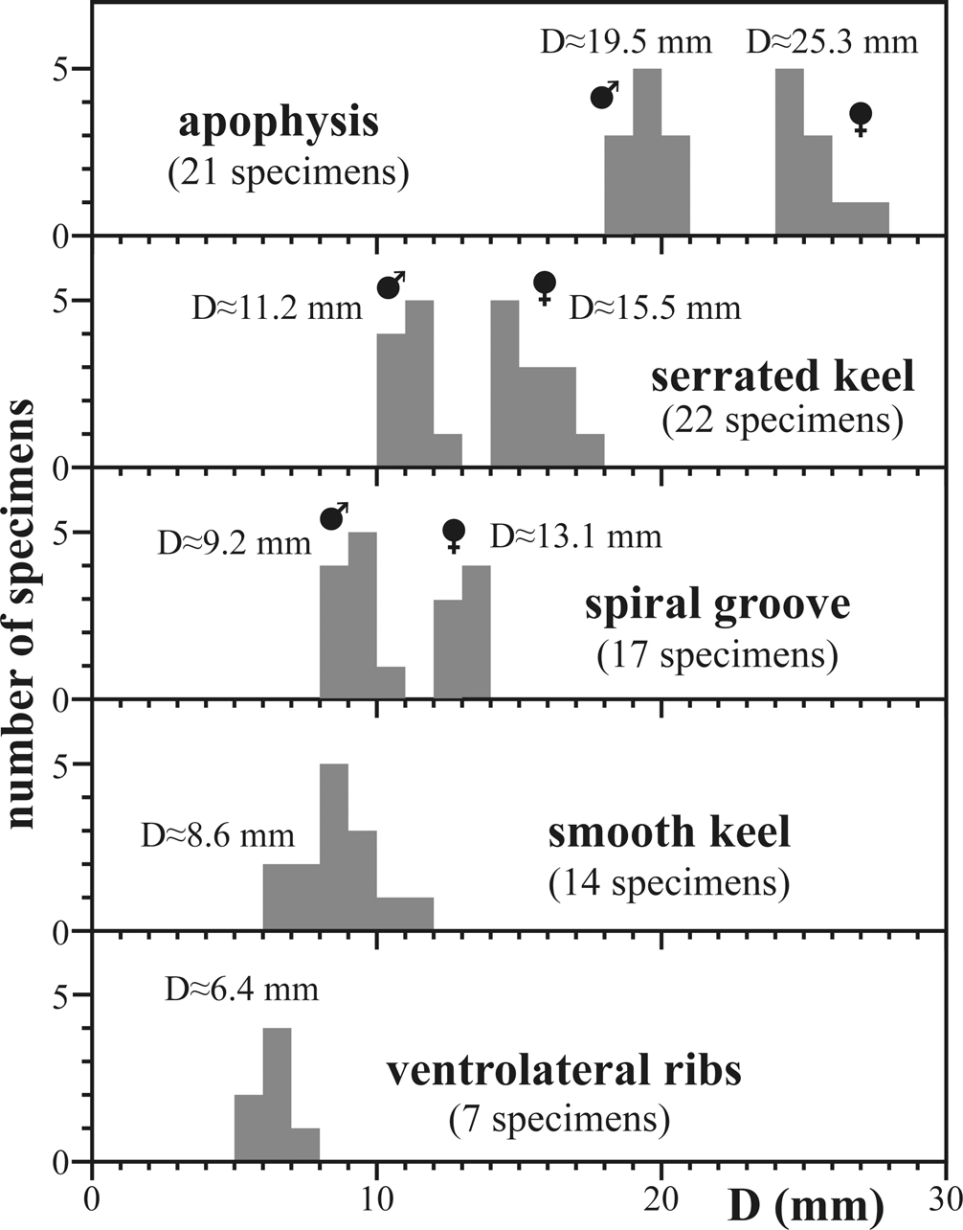
First occurrence of characteristic ornamentation patterns in *Salinites grossicostatum*. In macroconchs, the spiral groove, the serration of the keel and the apophysis appear at larger diameters.

The ornamentation shows five ontogenetic stages (Fig 3, Fig 10; see also [7], p. 27). The earliest ontogenetic stages are smooth with the exception of fine growth lines, which are, however, not visible on internal molds. Ventrolateral ribs appear at a diameter of 6.4 mm; they gradually increase in strength. A smooth keel is first seen at diameters of about 8.6 mm, which agrees with the description of Imlay [7] who noted a keel at diameters of about 10 mm. A spiral groove, situated at about mid-flank, first appears on internal molds at diameters of about 9.2 mm in microconchs and at about 13.1 mm in macroconchs. The keel becomes serrated at diameters of about 11.2 mm in microconchs and at about 15.5 mm in macroconchs. The serration gradually increases in strength and finally reaches the ventrolateral plain. An apophysis is first present at diameters of about 19.5 mm in microconchs and of about 25.3 mm in macroconchs; it is here interpreted to represent the adult stage, in accordance with previous interpretations of this character (e.g. [26]).

### Mature stage

The adult stage starts by the first appearance of an apophysis at D≈19.5 mm in microconchs and at D≈25.3 mm in macroconchs. It is characterized further by a distinct reduction of space between the septa, marking the final stage of their secretion (Fig 8). A similar pattern was previously documented in other ammonite taxa [2], [17], [27–29], among other authors. In the most completely preserved macroconchs (e.g. CPC-1411–1413) a slight decrease in the whorl overlap ratio is present at D>38 mm and the apophysis is consequently projecting ventrolaterally (Fig 3G, Fig 3H, Fig 3I), thus expressing a change in shell geometry. A crowding of growth lines, an additional characteristic of mature ammonites [29], is only visible in macroconch CPC-1413 (Fig 3I) at a diameter of 63 mm.

## Discussion

**Fig 11:**
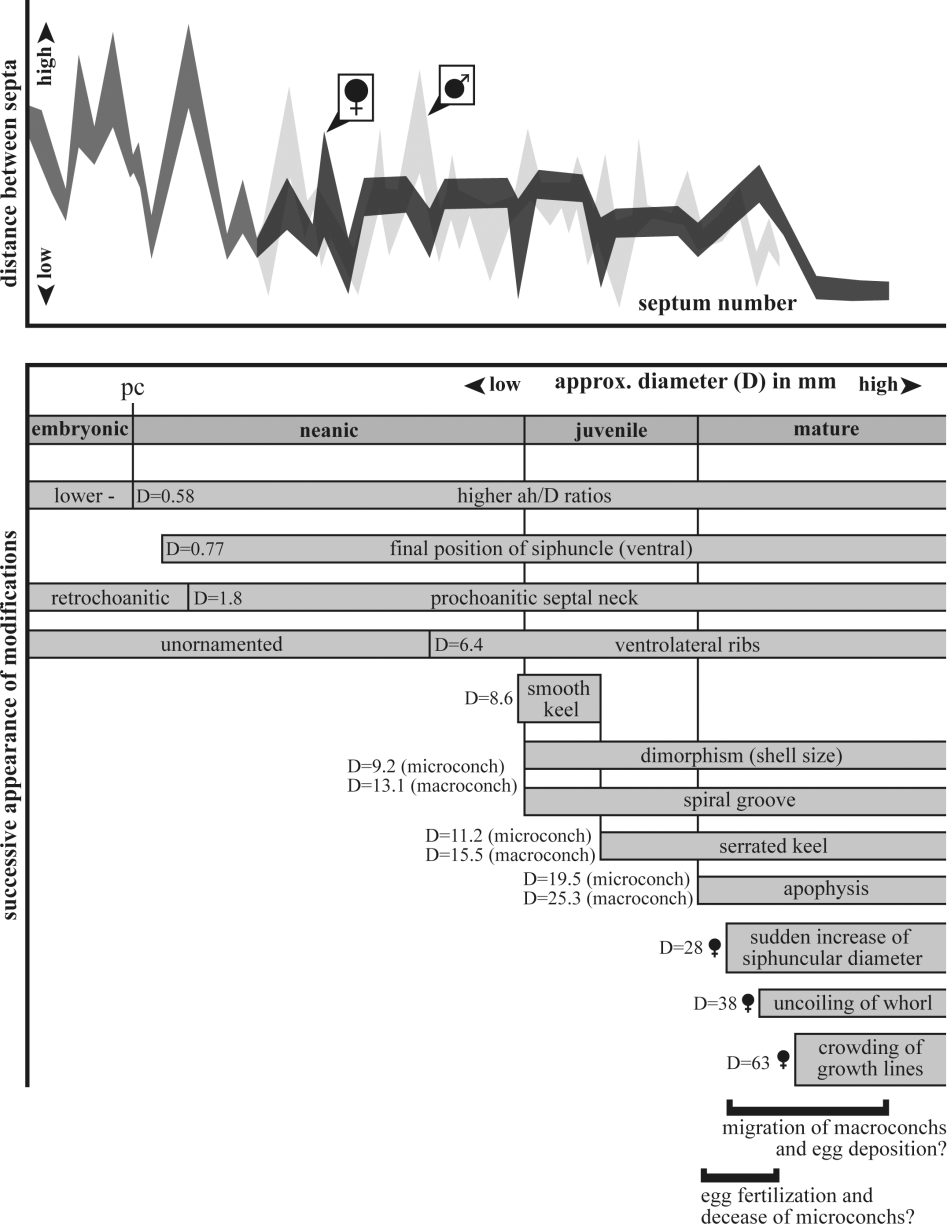
Ontogenetic reconstruction of *Salinites grossicostatum* based on external and internal characteristics. Sexual dimorphism and four major stages are recognized: embryonic, neanic, juvenile and mature. The diameter indicates the first occurrence of characteristic modifications.

Our analysis of the ontogeny of *Salinites grossicostatum* is based on 169 specimens collected from a single limestone layer at Puerto Piñones, Coahuila and assigned to the latest Tithonian “*Durangites* beds”. Results are summarized in Fig 11.

### Embryonic stage

Septal spaces during earliest ontogenetic stages agree in all four specimens analyzed here (micro- and macroconchs) and are marked by strong variations in septal spacing. Septal crowding during similarly early ontogenetic stages in nautiloids, bactritoids and ammonoids were interpreted to be related to hatching (e.g. [6], [30–33]).

### Neanic stage

The transition from an embryonic to neanic (early post-embryonic) stage is marked by a primary constriction at diameters of about 0.58 mm and by higher allometric growth in apertural height versus shell diameter, as also observed in other ammonite taxa [17], [34–36]. The primary constriction is followed by a sudden change from a retrochoanitic to prochoanitic septal neck in the first half of the second whorl. The transition from a backward-bending to a forward-bending septal neck is a common feature in early ontogenetic stages of ammonites and commonly detected in the first half of the second whorl (cf. [37]–[38]). In *S*. *grossicostatum* the siphuncle gradually shifts to a ventral position shortly after the primary constriction, thus coinciding with highest septal distances, similar to the nannoconch development in other planspiral ammonites [35], [39].

Numerous authors considered ammonite hatchlings to be planktonic (e.g. [40–45]). In that case, the highly variable septal angles observed in early ontogenetic stages of *S*. *grossicostatum* could reflect a change of life habit to the juvenile stage (cf. [6]). During the neanic development and up to about 30 septa, the ratio between the number of the septa and the diameter remains uniform for all specimens analyzed here.

### Juvenile stage

The juvenile stage of *S*. *grossicostatum* is represented by about equal numbers of micro- and macroconchs. This post-embryonic stage following the neanic stage and prior to sexual maturity, indicated by an apophysis, is characterized by low septal angles and a sexual dimorphism reflected in differential growth. (1) Microconchs show a higher variability in septal spacing than macroconchs, and (2) the ratio between the number of septa and the diameter, which was uniform during the neanic stage, splits up into two distinct lineages, a group of small-sized individuals (microconchs) and a second lineage of larger-sized specimens (macroconchs). Nevertheless, the general shape of growth curves remains similar for all individuals. Juvenile ammonoids have been considered to have almost uniform septal angles [39] and the juvenile *S*. *grossicostatum* makes no exception, with only a small variation in septal angles compared to earlier and later ontogenetic stages.

The shell remains unornamented until diameters of about 6.4 mm, where ventrolateral ribs first appear. A smooth keel develops at diameters of about 8.6 mm. A spiral groove was identified in microconchs at about 9.2 mm diameter and in macroconchs at about 13.1 mm. Serration of the keel is first present at diameters of about 11.2 mm in microconchs and at about 15.5 mm in macroconchs.

### Mature stage

Mature specimens of *S*. *grossicostatum* are notably less abundant than juveniles (26% compared to 74% juveniles) but the assemblage still presents an approximately equal number of mature micro- and macroconchs. Macroconchs of >53 mm diameter are relatively rare (8%) and even this small number may be biased due to preferential collection of larger specimens. Nevertheless, not a single individual was detected in the 45–53 mm size class. This bimodal size distribution differs from that of associated ammonites (e.g. *Durangites*, *Proniceras*, *Himalayites*) in which the percentage of adult specimens appears to be considerably higher and juveniles are rare. The size distribution pattern detected in *S*. *grossicostatum* may indicate a change in habitat preference of macroconchs, during a lapse following the accomplishment of sexual maturity and prior to the end of shell growth. Macroconchs may then have left the Puerto Piñones region, e.g. migration to shallow water environments for egg deposition, after mating and fertilization in the Puerto Piñones region. Concurrently, juveniles and adult microconchs of *S*. *grossicostatum* persisted in the area interpreted to represent outer shelf to uppermost slope environments [8]. Migration subsequent to egg fertilization is also seen in Recent *Nautilus*, where females choose shallow water environments for egg deposition [46]. After egg deposition, a small number (8%) of *S*. *grossicostatum* macroconchs with sizes of >53 mm diameter returned to the mating area at Puerto Piñones, possibly for a second mating event. Stable isotope data in the Jurassic ammonite *Cadoceras* [47] support this scenario as increasing δ^18^O values near the aperture (and thus during the latest ontogenetic stage) indicate migration to warm shallow water areas. This change in habitat was interpreted to be linked to spawning cycles.

The abundance of juveniles (D≈10 mm) at Puerto Piñones, but also at Sierra de Parras and Sierra de Jimulco originally described by Imlay [7], may indicate high mortality rates during early ontogenetic stages before the expression of sexual dimorphism, suggesting that *Salinites grossicostatum* reproduced early, quickly, and in large numbers, to ensure that at least some offspring of the population survive (r-selection). Ammonites associated with *S*. *grossicostatum* at Puerto Piñones are mostly represented by single adult individuals.

Siphuncular diameter analysis may provide additional evidence for mature macroconch migration. Generally, the siphuncular diameter gradually increases, although showing a negative allometry when compared with shell diameter and apertural height. Interestingly, a sudden increase in siphuncular diameter is detected in macroconchs during the late ontogenetic development (D≈28 mm), indicating a terminal stage. A negative allometry of the siphuncular diameter was previously documented in a variety of ammonites [35], [42], [48]–[49] and is also seen in modern *Nautilus* [17]. It was interpreted by Neige [17] as a reduction in the ability to maintain neutral buoyancy, while Ward [46] showed that in modern *Nautilus* the rate of filling of the cameral liquid is too slow to be involved in daily vertical movements. Nevertheless, filling was likely responsible for maintaining neutral buoyancy during early ontogenetic stages [17]. The sudden change from a low negative to a slightly higher negative allometry of the siphuncle, observed in adult macroconchs of *S*. *grossicostatum*, may thus represent a reaction to a lower growth rate of the organism [17] or/and changing habitat preferences, possibly for egg deposition.

In Recent *Nautilus*, a gradual reduction in septal space (septal crowding) characterizes the end of shell growth [27], [29]. This modification is either associated with an increased ability to trim buoyancy [50], or regarded as a compensation of a lower density of the soft body, resulting from the growth of reproductive organs [39]. Mature ammonoids, including mature *S*. *grossicostatum*, initially display increased septal angles followed by decreased angles over the last few septa [2], [27], [36], [51–54]. For example Tajika et al. [55] identified a decrease in volume (= spacing) over the last 5–7 septa in the Middle Jurassic *Normannites mitis*, while even higher numbers of crowded septa were detected in the Late Devonian *Pernoceras* (18 septa) and the Early Carboniferous *Ouaoufilalites* (14 septa; see [29], [56]). In *S*. *grossicostatum*, gradual septal crowding is identified for the final 12 septa in macroconchs, indicating a prolonged reduction of shell precipitation accompanying the end of growth (cf. [57]). Septal crowding is less expressed in microconchs and identified only for four septa. This disparity in the final septal spacing interval of micro- and macroconchs may indicate that the life span of macroconchs was longer and included a period long enough for migration and egg deposition. Even though, a reduction in septal spacing is not only found in adult ammonites but sometimes also occurs in preadults (e.g. [2], [57]); it was there interpreted to result from adverse ecological conditions. In *S*. *grossicostatum*, however, independent additional characters coincide with septal crowding and therefore confirm the end of growth, such as the presence of an apophysis in micro- and macroconchs, a smoothening of the ornamentation in macroconchs, a crowding of growth lines at the apophysis directly behind the terminal aperture, a sudden increase in siphuncular diameter in macroconchs, and a decrease in the whorl overlap ratio (uncoiling) of the shell (cf. [39] [58]–[59]).

### Seasonal changes and life span

The general trend in interseptal spacing in *S*. *grossicostatum* is highly variable during early ontogeny and is followed by a constant spacing including reduced spacing “events” (= crowding) and a reduced spacing marking the end of growth (Fig 11). Interseptal spacing changes were previously documented for the Jurassic ammonites *Hildoceras sublevisioni* [60] and *Creniceras renggeri* [17], where three stages in septum formation were identified. These stages may correspond to those observed in *S*. *grossicostatum*. In *Hildoceras sublevisioni*, there is also a tendency to reduced septal spacing “events”, similar to *S*. *grossicostatum*. In *Creniceras renggeri* the most closely spaced septa mark the end of growth, but this is not the case in *S*. *grossicostatum* and *Hildoceras sublevisioni* where the most closely spaced ribs were detected during earlier ontogenetic stages. Many environmental factors affect the rate of growth of marine organisms, e.g. temperature, diseases, food availability, pressure, light intensity, oxygen concentration, day length, and the abundance and kind of predators [39]. Among these parameters, temperature may have been a principal factor controlling the rate of growth. This is seen, for example, in modern *Sepia officinalis* from the English Channel [61] where the decrease in sea water temperatures is expressed in decelerated growth, a reduction in septal spacing, and an increase in the time of chamber formation [62]. Compared to Recent *Nautilus*, ammonoids show a wider variation in septal spacing [39]. This has partially been attributed to seasonality, with episodes of close septal spacing interpreted as slow-downs in growth associated with low temperatures [40], [48], [63]. Based on septal spacing, seasonality-influenced growth was inferred by Westermann [48] for middle Jurassic *Leioceras*, *Ludwigia* and *Sonninia*, leading to the identification of four to six possible annual cycles in these ammonites. Zakharov [63] recognized six episodes of reduced septal spacing in the Late Triassic *Pinacoceras* aff. *regiforme*, with about 80 septa and an estimated age of 7 years. Kraft et al. [2] concluded that premature septal crowding in ammonoids is related to disturbances of apertural shell growth for various reasons.

Based on our analysis of septal spacing we propose that seasonal environmental changes are evidenced in *Salinites grossicostatum*. Ten low septal angle events with almost constant amplitudes of fluctuation were identified during the ontogenetic development of this ammonite, all separated by eight to nine wide and regularly spaced septa. In *Nautilus*, these fluctuations in septal spacing are related to variations in the overall rate of growth and to the shape of shell and soft body ([39], and references therein), even though they are also seen in *Nautilus* kept in captivity [64].

During the latest Jurassic, the ancient Gulf of Mexico was repeatedly affected by phases of upwelling of cold and nutrient rich bottom waters, evidenced by intermittent upwelling-related phosphatic layers in the Kimmeridgian–Tithonian La Casita and La Caja formations of northeastern Mexico (e.g. [65]). This upwelling system would have provided favorable nutrient-rich environmental conditions for most of the year and only have ceased during major monsoonal phases, similar to modern ocean systems [66]. The debilitated upwelling temporarily lowered food availability. The abundance of juvenile *Salinites grossicostatum* in the assemblage may therefore relate to these episodes while surviving specimens responded to these phases with the production of low septal angles. 8.5 similar spaced septa are present between these septal “events”, indicating that *S*. *grossicostatum* built up about 9.5 septa per year. 97 septa were identified in the largest specimen analyzed (CPC-1214), which would correspond to a life span of 10 years. Maturity was reached after 81 septa, or 8.5 years, compared to 10 years in Recent *Nautilus* [67].

## Conclusions

Based on a collection of 169 specimens of juveniles and adults we document the ontogenetic history of the latest Tithonian ammonite *Salinites grossicostatum* ([7]). The protoconch is small and subelliptical in outline, comparable to other Jurassic ammonites (cf. [17]). The transition between the embryonic and the neanic stages is marked by a primary constriction at approximately D = 0.58 mm diameter and by a break in allometric growth (e.g. ratio between apertural height and diameter). The initial position of the siphuncle is central to subcentral but gradually shifts ventrally at about one whorl after the end of the protoconch. The septal neck is retrochoanitic and becomes prochoanitic, possibly during the neanic stage. A sexual dimorphism is expressed from the neanic stage by differential growth rates and different septal spacing trends. Three general stages of interseptal spacing are identified (1) Septal spacing during early ontogenetic stages is marked by significant fluctuations. This phase is followed (2) by a relatively stable septal growth in macroconchs and a variable stage in microconchs, indicating a change in habitat preferences for juveniles. The final mature phase (3) is marked by a gradual decrease in interseptal spacing and marks the end of shell secretion, similar to other ammonites. At least ten septal “events” are present and interpreted as seasonally-caused periods of ecological stress. This scenario implies that the formation of new septa was controlled by time, rather than by the distances from the proceeding septum (cf. [2], [50], [68]). *Salinites grossicostatum* possibly responded to ecological stress (e.g. annual environmental changes) with the production of low septal angles. We suggest that the oceanic upwelling system known from the La Caja Ocean in the area periodically (e.g. annually) ceased or collapsed, leading to reduced food availability, temperature changes etc. The ammonite responded to this stress with decelerated shell growth and thus septal crowding. These seasonal changes would also explain the high mortality rates as indicated by the abundance of juvenile specimens with shell sizes similar to those of septal crowding in survivors. 8.5 evenly spaced septa are present between these septal “events”, which suggests that *S*. *grossicostatum* built up about 9.5 septa per year. In that case large specimens reached a life span of 10 years and maturity was reached after 81 septa, or 8.5 years.

Six ornamentational stages are differentiated. During early ontogeny, *S*. *grossicostatum* is unornamented. This phase is followed by the subsequent appearance of ventrolateral ribs, a smooth keel, a spiral groove, and by the serration of this keel. Maturity is reached at approximately 19.5 mm diameter in microconchs and 25.3 mm in macroconchs, and is marked by the appearance of an apophysis and a reduction of septal spacing. Additional mature characteristics are expressed in macroconchs, including smoothening of surface ornamentation, increase in siphuncular diameter, reduced whorl overlap ratio, and the crowding of growth lines on the apophysis.

Mortality rates were high during early ontogenetic stages before the expression of sexual dimorphism, suggesting that *Salinites grossicostatum* was an r-selective species. Size distribution and a sudden increase in siphuncular diameter in macroconchs may indicate changing habitat preferences of macroconchs during an interval after reaching sexual maturity and before their end of growth. Macroconchs may have left the mating area at Puerto Piñones to reach shallow water areas for egg deposition.
